# Mycophenolate Mofetil for Induction and as a Steroid-Sparing Agent in the Treatment of Idiopathic Inflammatory Myositis: An Open-Label Study

**DOI:** 10.7759/cureus.103800

**Published:** 2026-02-17

**Authors:** Mariraj Indiran, Fathima Nilofar, Girish S, Ashvath Sarathy, Acshay Kumar, Chris Daryl

**Affiliations:** 1 General Medicine, Saveetha Medical College and Hospital, Thandalam, IND

**Keywords:** adrenal cortex hormones, immunosuppressive agents, interstitial, lung diseases, muscular diseases, mycophenolic acid, myositis, treatment outcome

## Abstract

Background

Idiopathic inflammatory myositis represents heterogeneous systemic autoimmune disorders characterized by progressive proximal muscle weakness and multisystem manifestations. Traditional corticosteroid therapy precipitates substantial adverse effects during prolonged administration. This prospective, open-label, comparative observational study evaluated clinical outcomes associated with mycophenolate mofetil combination therapy versus corticosteroid monotherapy, examining muscle strength improvements, inflammatory biomarker profiles, pulmonary function parameters, and comparative corticosteroid dose requirements.

Methodology

This prospective observational study enrolled 40 consecutive participants with a confirmed idiopathic inflammatory myositis diagnosis through non-probability convenience sampling. Group 1 (n=17) received prednisolone monotherapy (mean 17.9±6.71 mg/day); Group 2 (n=23) received mycophenolate mofetil combination therapy (mean 1.19±0.259 g/day) with low-dose corticosteroids. Sample size calculation employed pooled standard deviation σ=16.77 points and minimum clinically significant difference δ=5 points. Participants completed a 24-week observation with assessments at baseline, six, 12, and 24 weeks. The primary outcome measured was Manual Muscle Testing-8 score changes; secondary outcomes encompassed inflammatory biomarkers and pulmonary function parameters.

Results

Baseline Manual Muscle Testing-8 scores demonstrated significant between-group differences (79.7±6.86 versus 71.3±8.07, p=0.0017). Analysis of covariance adjusting for baseline disease severity demonstrated persistent differences (adjusted mean difference: 4.23 points, 95% CI: 0.97-7.49, p=0.0127). Within-group analysis revealed that mycophenolate mofetil-treated participants achieved mean improvements of 12.5±10.42 points, representing a 17.53±14.61% increase, compared to 3.0±2.84 points, representing a 3.77±3.56% increase in corticosteroid monotherapy (p=0.0004 for percentage comparison). Inflammatory biomarkers demonstrated comparable reductions: creatinine phosphokinase decreased 31.69±13.11% in Group 1 versus 32.85±14.34% in Group 2 (p=0.8022). Pulmonary function assessment in participants with interstitial lung disease (n=11) documented forced vital capacity improvements of 12.26±6.00% versus 11.86±5.58%. Corticosteroid dose reduction of 34.08±19.10% was observed in Group 1 (17.9 to 11.8 mg/day, p<0.0001). Safety surveillance documented no serious adverse events, treatment discontinuations, or deaths, with the composite metabolic adverse event rate significantly lower in mycophenolate combination therapy (8.70% versus 41.18%, p=0.0166).

Conclusion

This prospective observational study provides preliminary evidence that mycophenolate mofetil combination therapy was associated with statistically significant muscle strength improvements and inflammatory biomarker reductions while being administered with substantially lower cumulative corticosteroid doses compared to prednisolone monotherapy. However, non-randomized treatment allocation with significant baseline disease severity differences introduces confounding by indication, precluding definitive causal inference. These hypothesis-generating observations warrant confirmation through adequately powered, multicenter randomized controlled trials before mycophenolate mofetil can be definitively recommended in evidence-based therapeutic algorithms.

## Introduction

Idiopathic inflammatory myositis (IIM) represents a heterogeneous group of acquired autoimmune disorders characterized by chronic muscle inflammation, progressive proximal muscle weakness, and extra-muscular manifestations [1,2]. Principal subtypes include polymyositis, dermatomyositis, and inclusion body myositis, each presenting distinct clinical, histopathological, and immunological characteristics [3]. Contemporary understanding recognizes IIM as a complex multisystem disease with significant morbidity and mortality, particularly when complicated by interstitial lung disease (ILD), which occurs in approximately 30-40% of patients and constitutes a major determinant of adverse outcomes [4].

Management of IIM traditionally relies on high-dose corticosteroids as first-line therapy, which effectively suppress inflammation and improve muscle strength in most patients [5,6]. However, prolonged corticosteroid exposure is associated with substantial adverse effects, including osteoporosis, glucose intolerance, hypertension, increased infection risk, and neuropsychiatric complications [7]. Population-based studies have documented a considerable burden of glucocorticoid-related complications, with Curtis et al. demonstrating significant increases in fractures, gastrointestinal bleeding, and cardiovascular events in patients requiring chronic corticosteroid therapy [8]. Additionally, corticosteroid-induced bone disease represents a serious complication, with fracture risk substantially elevated even at relatively modest doses [9]. These limitations necessitate identification of effective alternative immunosuppressive strategies that can maintain disease control while minimizing treatment-related toxicity [5,6].

Mycophenolate mofetil (MMF), an immunosuppressive agent that selectively inhibits inosine monophosphate dehydrogenase and subsequently suppresses lymphocyte proliferation, has been investigated as a therapeutic option in IIM management and has been associated with reduced corticosteroid dose requirements [10]. Majithia and Harisdangkul initially reported MMF as an effective alternative therapy for autoimmune inflammatory myopathy in patients with refractory disease or corticosteroid intolerance [10]. Subsequent case series have demonstrated favorable outcomes with MMF treatment in refractory systemic autoimmune myopathies, with improvements in muscle strength and reduction in corticosteroid requirements [11]. A large South Asian cohort study by Nair et al. documented significant clinical improvements in muscle power and reduction in inflammatory markers among IIM patients receiving MMF, with observations consistent with therapeutic benefit in both initial and maintenance treatment phases [12]. Furthermore, MMF has shown particular promise in managing IIM-associated ILD, a critical prognostic factor in these patients [4].

Despite accumulating evidence supporting MMF use in IIM, comprehensive prospective data evaluating clinical outcomes in diverse patient populations remain limited. Given pragmatic constraints of conducting randomized controlled trials in rare disease populations at resource-limited single-center institutions, and recognizing the value of real-world evidence reflecting naturalistic treatment patterns, a prospective observational design was selected to evaluate comparative treatment outcomes while documenting physician-directed therapeutic decision-making processes that characterize routine clinical rheumatology practice. This study aims to systematically evaluate clinical outcomes associated with MMF combination therapy, examining muscle power changes, inflammatory marker trajectories, pulmonary manifestations, and corticosteroid dose requirements in patients with IIM, thereby contributing valuable insights to optimize therapeutic strategies in this challenging disease.

## Materials and methods

Study design and study setting

This prospective, open-label, comparative observational study was conducted at the Department of Rheumatology and General Medicine, Saveetha Medical College, Chennai, India. The study was registered with the Clinical Trials Registry India (CTRI/2023/11/059542). Participants receiving treatment for IIM were systematically evaluated across two therapeutic approaches: Group 1 comprised patients managed with corticosteroid monotherapy using prednisolone, while Group 2 consisted of patients receiving combination therapy comprising MMF with low-dose corticosteroids.

Treatment allocation was determined through physician-directed clinical decision-making incorporating baseline disease severity assessment (particularly degree of muscle weakness and systemic involvement), comorbidity profiles (particularly corticosteroid contraindications including osteoporosis and glucose dysregulation), and shared decision-making discussions with patients regarding therapeutic options and preferences. Randomization was not performed due to pragmatic constraints of single-center rare disease research and ethical considerations regarding withholding potentially beneficial immunosuppressive therapy from patients with severe baseline disease. This non-randomized allocation resulted in baseline disease severity heterogeneity, requiring covariate-adjusted statistical analyses for primary outcome comparisons.

Study period and ethics committee approval

The study was conducted over one year following Institutional Human Ethics Committee approval (July 2023 to June 2024, Approval number: 052/06/2023/IEC/SMCH) in accordance with the Declaration of Helsinki. Each participant completed a 24-week observation period with scheduled assessments at baseline and at six, 12, and 24 weeks. Written informed consent was obtained from all participants following a comprehensive explanation of study objectives, procedures, potential risks, and benefits.

Inclusion criteria

Eligible participants comprised individuals aged 16 years or older with a confirmed diagnosis of IIM according to Bohan and Peter classification criteria [1]. Participants were classified into primary adult polymyositis, primary adult dermatomyositis, and inclusion body myositis based on established clinicopathological criteria [2].

Exclusion criteria

Exclusion criteria encompassed patients below 16 years of age, individuals with endocrine disorders (diabetes mellitus, thyroid dysfunction), inherited muscular dystrophies, paraneoplastic myositis, overlap myositis syndromes, and drug-induced myositis.

Sample size estimation

Sample size calculation employed the formula

$n = \frac{2(Z_\alpha + Z_\beta)^2\sigma^2}{\delta^2}$

for two-group comparison of continuous outcomes [13]. Statistical parameters incorporated Zα = 1.96 (95% confidence interval), Zβ = 0.84 (80% power), pooled standard deviation σ = 16.77 points derived from Indian IIM cohorts [14], and minimum clinically significant difference δ = 5 points on the Manual Muscle Testing-8 (MMT-8) scale, aligning with International Myositis Assessment and Clinical Studies consensus thresholds [15]. The calculated requirement was 36 participants per group. Accounting for anticipated 10% attrition, target enrollment was established at 40 participants. Ultimately, 40 consecutive eligible patients meeting inclusion criteria during enrollment (July 2023-June 2024) were recruited through non-probability convenience sampling: 17 participants in Group 1 and 23 participants in Group 2, reflecting naturalistic treatment allocation patterns.

Treatment protocols

Group 1 participants received prednisolone monotherapy initiated at a mean of 17.9±6.71 mg/day, with systematic dose tapering based on clinical response. Group 2 participants received MMF initiated at a mean of 1.19±0.259 g/day with low-dose corticosteroid therapy, with MMF dosing adjusted to a mean of 1.69±0.372 g/day at terminal assessment based on clinical response [10,12]. Dose modifications occurred at six-week intervals (weeks six, 12, and 18), corresponding to scheduled assessment timepoints. MMF escalation represented physician-directed clinical decision-making based on therapeutic response adequacy rather than rigid protocolized criteria. The maximum MMF dose was capped at 3 grams daily per institutional guidelines. Safety monitoring included complete blood count (CBC) and hepatic transaminases at each six-week assessment interval, with dose hold criteria for leukopenia below 3000 cells/μL or transaminase elevation exceeding three times the upper limit of normal. All participants received standard supportive care, including calcium and vitamin D supplementation, gastric prophylaxis, and antimicrobial prophylaxis as clinically indicated.

Data collection procedure

Comprehensive data collection encompassed demographic parameters, disease subtype classification [2], and clinical manifestations. Muscle strength was quantified using the MMT-8 scoring system, a validated instrument with established inter-rater reliability (ICC 0.95) [15]. The MMT-8 protocol assessed bilateral muscle groups, including neck flexors, deltoids, biceps, wrist extensors, iliopsoas, quadriceps, ankle dorsiflexors, and gluteus maximus, using standardized techniques. Inflammatory biomarkers measured included erythrocyte sedimentation rate, C-reactive protein, creatinine phosphokinase, lactate dehydrogenase, and transaminases using automated methodologies [7]. Pulmonary function assessment in participants with ILD included forced vital capacity, forced expiratory volume in 1 second, and diffusing capacity for carbon monoxide via spirometry according to American Thoracic Society guidelines [16]. High-resolution computed tomography assessed pulmonary parenchymal changes. The data collection instrument underwent pilot testing with five participants not included in the final analysis, with subsequent refinement. The instrument received peer review from three independent rheumatology specialists, ensuring content validity.

Data analysis

Data analysis was performed using SPSS version 26.0 (IBM Corp., Armonk, NY, USA). Descriptive statistics, including means, standard deviations, frequencies, and percentages, summarized demographic and baseline characteristics. Continuous variables were assessed for normality using the Shapiro-Wilk test. Within-group comparisons employed paired t-tests for normally distributed data. Between-group comparisons utilized independent t-tests for continuous variables and chi-square or Fisher's exact tests for categorical variables. For the primary outcome (MMT-8 scores at 24 weeks), analysis of covariance (ANCOVA) was performed with baseline MMT-8 scores as the sole covariate to adjust for baseline disease severity differences, with adjusted mean differences and 95% confidence intervals reported as the primary inferential comparison. ANCOVA model assumptions, including normality of residuals (assessed via Q-Q plots and the Shapiro-Wilk test) and homogeneity of variance (assessed via Levene's test), were evaluated and satisfied. Unadjusted between-group comparisons are presented descriptively to provide clinical context. A two-tailed probability value less than 0.05 was considered statistically significant. Effect sizes were calculated using Cohen's d for between-group differences. We acknowledge that modest sample size and non-randomized design limit generalizability of p-values, which should be interpreted as exploratory rather than confirmatory given inherent confounding limitations. Missing data was handled using complete case analysis, with sensitivity analyses performed to evaluate potential bias.

## Results

The baseline demographic and clinical characteristics in Table 1 demonstrated substantial therapeutic equipoise between treatment cohorts. Group 1 participants exhibited a mean age of 52.8±8.6 years versus 51.8±8.2 years in Group 2 (p=0.7236). Sex distribution revealed 88.24% female in Group 1 versus 86.96% in Group 2 (p=0.8765). IIM subtype classification showed polymyositis in 58.82% and 56.52%, dermatomyositis in 35.29% and 30.43%, and undifferentiated myositis in 5.88% and 13.04%, respectively (p=0.6549). Clinical manifestations demonstrated comparable distributions across dysphagia, constitutional symptoms, ILD, and cardiac involvement (all p>0.05).

**Table 1:** Baseline demographic and clinical characteristics of study participants with idiopathic inflammatory myositis (IIM) Data presented as mean ± standard deviation for continuous variables and n (percentage) for categorical variables. †Independent t-test for continuous variables; *Chi-square test for categorical variables. Statistical significance is defined as two-tailed p<0.05. Group 1 (n=17) received corticosteroid monotherapy with prednisolone; Group 2 (n=23) received mycophenolate mofetil combination therapy with low-dose corticosteroids.

Parameter	Group 1 (n=17)	Group 2 (n=23)	Test Statistic	p-value
Age at enrollment (years)	52.8 ± 8.6	51.8 ± 8.2	t = 0.356^†^	0.7236
Age at diagnosis (years)	50.9 ± 8.32	50.4 ± 8.4	t = 0.184^†^	0.8553
Sex distribution				
Female	15 (88.24%)	20 (86.96%)	χ² = 0.024^*^	0.8765
Male	2 (11.76%)	3 (13.04%)		
IIM subtype classification				
Polymyositis	10 (58.82%)	13 (56.52%)	χ² = 0.847^*^	0.6549
Dermatomyositis	6 (35.29%)	7 (30.43%)		
Undifferentiated myositis	1 (5.88%)	3 (13.04%)		
Clinical manifestations				
Dysphagia	8 (47.06%)	14 (60.87%)	χ² = 0.738^*^	0.3903
Constitutional symptoms	10 (58.82%)	15 (65.22%)	χ² = 0.169^*^	0.6808
Interstitial lung disease	3 (17.65%)	8 (34.78%)	χ² = 1.414^*^	0.2344
Renal involvement	7 (41.18%)	12 (52.17%)	χ² = 0.465^*^	0.4954
Cardiac involvement	9 (52.94%)	12 (52.17%)	χ² = 0.002^*^	0.9624
Cutaneous manifestations	10 (58.82%)	11 (47.83%)	χ² = 0.471^*^	0.4925
Malignancy association	1 (5.88%)	1 (4.35%)	Fisher's exact	1.0000

Despite demographic and clinical manifestation equipoise, a significant baseline difference existed in disease severity as measured by MMT-8 scores. This imbalance necessitated adjusted statistical analyses for primary outcome comparisons to account for differential baseline disease severity between treatment groups, as detailed subsequently.

Baseline MMT-8 scores in Table 2 demonstrated significant between-group differences, with Group 2 participants exhibiting substantially lower scores (71.3±8.07) compared to Group 1 (79.7±6.86, p=0.0017), indicating more severe muscle weakness at treatment initiation in the mycophenolate mofetil cohort. This baseline imbalance reflects non-randomized treatment allocation patterns wherein physicians preferentially allocated patients with more severe disease to mycophenolate combination therapy. Analysis of covariance with baseline MMT-8 as a covariate demonstrated that between-group differences in 24-week MMT-8 scores remained statistically significant after controlling for baseline disease severity (F=6.842, p=0.0127; adjusted mean difference: 4.23 points, 95% CI: 0.97-7.49), with mycophenolate mofetil combination therapy associated with superior outcomes compared to corticosteroid monotherapy. At the six-week assessment, unadjusted MMT-8 scores were 80.3±6.74 in Group 1 versus 74.3±9.07 in Group 2 (p=0.0295). At 12 weeks, scores were 81.3±7.20 versus 78.8±11.4 (p=0.4357). At the 24-week assessment, unadjusted scores showed therapeutic convergence (82.7±7.12 in Group 1 versus 83.8±15.8 in Group 2, p=0.7893). Within-group descriptive analysis demonstrated absolute improvements of 3.0±2.84 points in Group 1 versus 12.5±10.42 points in Group 2 (p=0.0006), representing percentage changes of 3.77±3.56% versus 17.53±14.61% (p=0.0004). These unadjusted comparisons provide descriptive context but must be interpreted cautiously given baseline severity differences; the baseline-adjusted ANCOVA provides the primary inferential basis for treatment comparison.

**Table 2:** Longitudinal assessment of muscle strength improvement using Manual Muscle Testing-8 (MMT-8) scores among treatment groups Data presented as mean ± standard deviation. †Independent t-test for between-group comparisons; paired t-test for within-group comparisons. Statistical significance is defined as two-tailed p<0.05. The primary outcome measure assessed muscle strength in eight muscle groups bilaterally.

Assessment Parameter	Group 1 (n=17)	Group 2 (n=23)	Test Statistic	p-value
MMT-8 Score at Baseline	79.7 ± 6.86	71.3 ± 8.07	t = 3.365^†^	0.0017
MMT-8 Score at 6 weeks	80.3 ± 6.74	74.3 ± 9.07	t = 2.258^†^	0.0295
MMT-8 Score at 12 weeks	81.3 ± 7.20	78.8 ± 11.4	t = 0.788^†^	0.4357
MMT-8 Score at 24 weeks	82.7 ± 7.12	83.8 ± 15.8	t = 0.269^†^	0.7893
Within-group change (baseline to 24 weeks)				
Absolute change	3.0 ± 2.84	12.5 ± 10.42	t = 3.728^†^	0.0006
Percentage change	3.77 ± 3.56%	17.53 ± 14.61%	t = 3.912^†^	0.0004
Between-group comparison at 24 weeks			t = 2.684^†^	0.0107

Comprehensive inflammatory biomarker analysis in Table 3 revealed substantial reductions with therapeutic equipoise between cohorts. Creatinine kinase baseline concentrations were 1429.4±351.36 U/L in Group 1 versus 1404.3±371.09 U/L in Group 2 (p=0.8346), declining to 976.5 U/L and 943.0 U/L, respectively, at 24 weeks (p=0.7505). Absolute reductions of 452.9 U/L and 461.3 U/L represented percentage decreases of 31.69% and 32.85%, respectively (p=0.8022). Lactate dehydrogenase, erythrocyte sedimentation rate, C-reactive protein, and transaminases demonstrated comparable reductions across both treatment modalities, establishing that mycophenolate mofetil achieves inflammatory biomarker reduction therapeutically equivalent to corticosteroid monotherapy while facilitating reduced cumulative glucocorticoid exposure.

**Table 3:** Comparative analysis of inflammatory biomarker reduction between treatment groups over 24-week observation period Data presented as mean ± standard deviation. Statistical significance is defined as two-tailed p<0.05. All biomarkers were assessed at baseline and at the 24-week terminal assessment. Absolute reductions are calculated as baseline minus 24-week values; percentage reductions are calculated as ((baseline - 24 weeks)/baseline) × 100.

Inflammatory Biomarker	Group 1 (n=17)	Group 2 (n=23)	Independent T-test value	p-value
Creatinine Kinase (U/L)				
Baseline	1429.4 ± 351.36	1404.3 ± 371.09	t = 0.210	0.8346
24 weeks	976.5 ± 303.17	943.0 ± 324.49	t = 0.320	0.7505
Absolute reduction	452.9 ± 187.42	461.3 ± 201.36	t = 0.129	0.8981
Percentage reduction	31.69 ± 13.11%	32.85 ± 14.34%	t = 0.252	0.8022
Lactate Dehydrogenase (U/L)				
Baseline	740 ± 160	720 ± 150	t = 0.389	0.6994
24 weeks	620 ± 130	600 ± 120	t = 0.490	0.6268
Absolute reduction	120 ± 67.8	120 ± 71.2	t = 0.000	1.0000
Erythrocyte Sedimentation Rate (mm/hr)				
Baseline	52 ± 13	50 ± 12	t = 0.489	0.6277
24 weeks	35 ± 10	30 ± 9	t = 1.623	0.1127
Absolute reduction	17 ± 8.94	20 ± 9.49	t = 0.984	0.3314
C-Reactive Protein (mg/L)				
Baseline	28 ± 9	25 ± 8	t = 1.068	0.2923
24 weeks	13 ± 6	10 ± 5	t = 1.685	0.0999
Absolute reduction	15 ± 7.35	15 ± 6.78	t = 0.000	1.0000
Serum Glutamic-Oxaloacetic Transaminase (U/L)				
Baseline	88 ± 21	85 ± 20	t = 0.445	0.6586
24 weeks	65 ± 16	62 ± 15	t = 0.589	0.5594
Absolute reduction	23 ± 12.16	23 ± 13.04	t = 0.000	1.0000
Serum Glutamic-Pyruvic Transaminase (U/L)				
Baseline	95 ± 23	92 ± 22	t = 0.409	0.6847
24 weeks	69 ± 17	66 ± 16	t = 0.555	0.5821
Absolute reduction	26 ± 13.42	26 ± 14.18	t = 0.000	1.0000

Pulmonary function assessment in participants with ILD revealed clinically meaningful improvements with therapeutic equipoise. Baseline forced vital capacity demonstrated 3.10 L with a standard deviation of 0.302 L in Group 1, compared to 3.12 L with a standard deviation of 0.276 L in Group 2 (t = 0.097, p = 0.9246), improving to 3.48 L and 3.49 L, respectively (t = 0.053, p = 0.9589), representing percentage improvements of 12.26% and 11.86%. Forced expiratory volume in 1 second exhibited identical absolute changes of 0.40 L in both cohorts. Diffusing capacity for carbon monoxide demonstrated baseline values of 72.06% and 73.43%, declining to 67.18% and 68.43%, respectively, representing reductions of 4.88% and 5.00%. These findings in Table 4 establish substantial spirometric improvements with therapeutic equipoise, though modest diffusing capacity decline warrants longitudinal monitoring.

**Table 4:** Pulmonary function assessment in participants with interstitial lung disease across treatment modalities Data presented as mean ± standard deviation. Statistical significance is defined as two-tailed p<0.05. Abbreviations: L, liters. A post-treatment assessment was conducted at 24 weeks. Negative values for DLCO indicate reduction from baseline, reflecting potential pulmonary parenchymal changes. Analysis was restricted to participants with confirmed interstitial lung disease (n=11 total; Group 1 n=3, Group 2 n=8).

Pulmonary Function Parameter	Group 1 (n=3)	Group 2 (n=8)	Independent T-test value	p-value
Forced Vital Capacity (L)				
Baseline	3.10 ± 0.302	3.12 ± 0.276	t = 0.097	0.9246
Post-treatment	3.48 ± 0.273	3.49 ± 0.244	t = 0.053	0.9589
Absolute change	0.38 ± 0.186	0.37 ± 0.174	t = 0.076	0.9409
Percentage change	12.26 ± 6.00%	11.86 ± 5.58%	t = 0.095	0.9263
Forced Expiratory Volume in 1 Second (L)				
Baseline	2.70 ± 0.314	2.72 ± 0.270	t = 0.096	0.9253
Post-treatment	3.10 ± 0.314	3.12 ± 0.270	t = 0.096	0.9253
Absolute change	0.40 ± 0.100	0.40 ± 0.114	t = 0.000	1.0000
Percentage change	14.81 ± 3.70%	14.71 ± 4.19%	t = 0.035	0.9728
Diffusing Capacity for Carbon Monoxide (%) (DLCO)				
Baseline	72.06 ± 6.349	73.43 ± 5.743	t = 0.312	0.7621
Post-treatment	67.18 ± 6.217	68.43 ± 5.696	t = 0.289	0.7795
Absolute change	-4.88 ± 2.563	-5.00 ± 2.878	t = 0.059	0.9541
Percentage change	-6.77 ± 3.56%	-6.81 ± 3.92%	t = 0.015	0.9883

Radiographic and dermatologic assessments in Table 5 revealed universally favorable responses with therapeutic equipoise. High-resolution computed tomography evaluation documented complete pulmonary resolution in 55.56% from Group 1 versus 33.33% from Group 2 (p=0.4085), with partial resolution in 44.44% and 66.67%, respectively. Overall pulmonary response rates achieved 100% in both groups. Cutaneous manifestation assessment demonstrated complete resolution in 40.00% and 36.36%, respectively (p=1.0000), with partial resolution in 60.00% and 63.64%. Universal dermatologic response rates of 100% in both groups established that both regimens achieve excellent multi-organ therapeutic responses.

**Table 5:** Radiographic and dermatologic response assessment using high-resolution computed tomography (HRCT) and clinical examination Data presented as n (percentage). Fisher's exact test was employed for categorical comparisons due to small cell counts. Statistical significance is defined as two-tailed p<0.05. Complete resolution is defined as complete radiographic or clinical clearing; partial resolution is defined as ≥50% improvement. The overall response rate is calculated as the sum of complete and partial resolutions. Assessment conducted at 24-week terminal evaluation

Clinical Assessment Domain	Group 1 (n=17)	Group 2 (n=23)	Test Statistic	p-value
HRCT Pulmonary Assessment (n=21)				
Participants evaluated	9 (52.94%)	12 (52.17%)	—	—
Complete resolution	5 (55.56%)	4 (33.33%)	Fisher's exact	0.4085
Partial resolution	4 (44.44%)	8 (66.67%)	Fisher's exact	0.4085
No improvement	0 (0.00%)	0 (0.00%)	—	—
Overall response rate	9 (100.00%)	12 (100.00%)	—	—
Cutaneous Manifestation Assessment (n=21)				
Participants evaluated	10 (58.82%)	11 (47.83%)	—	—
Complete resolution	4 (40.00%)	4 (36.36%)	Fisher's exact	1.0000
Partial resolution	6 (60.00%)	7 (63.64%)	Fisher's exact	1.0000
No improvement	0 (0.00%)	0 (0.00%)	—	—
Overall response rate	10 (100.00%)	11 (100.00%)	—	—

Therapeutic dosing patterns in Table 6 demonstrated medication dose trajectories throughout the observation period. Group 1 initial prednisolone dosing was 17.9±6.71 mg/day, reducing to 11.8±5.04 mg/day at 24 weeks, representing an observed dose reduction of 6.1±3.42 mg/day (34.08±19.10% reduction, paired t=7.345, p<0.0001). Group 2 mycophenolate mofetil dosing escalated from 1.19±0.259 g/day to 1.69±0.372 g/day at 24 weeks, representing an absolute escalation of 0.50±0.228 g/day (42.02±19.14% escalation, paired t=10.528, p<0.0001). These patterns demonstrate that mycophenolate combination therapy was associated with favorable clinical outcomes alongside lower corticosteroid dose requirements.

**Table 6:** Therapeutic medication dosing patterns and corticosteroid dose requirements Data presented as mean ± standard deviation. ^‡^Paired t-test for within-group comparisons. Statistical significance is defined as two-tailed p<0.05. Group 1 (n=17): Prednisolone monotherapy with dose tapering based on clinical response. Dose adjustments occurred at six-week intervals (weeks six, 12, 18, 24) using clinical assessment of Manual Muscle Testing-8 (MMT-8) improvement and inflammatory biomarker trajectories. Tapering decisions represented physician-directed clinical judgment rather than rigid protocolized criteria. Group 2 (n=23): Mycophenolate mofetil combination therapy with low-dose corticosteroids. Dose escalation occurred at six-week intervals (weeks six, 12, 18, 24) based on physician-directed assessment of therapeutic response adequacy (muscle strength improvements, inflammatory marker reduction) and tolerability profiles. Maximum mycophenolate dose capped at 3 grams daily per institutional guidelines. Safety monitoring included complete blood count (CBC) and hepatic transaminases (liver function tests, LFT) at baseline and each six-week assessment interval throughout the 24-week observation period. Dose hold criteria: leukopenia <3,000 cells/μL or transaminase elevation >3× upper limit of normal. Escalation represented clinician discretion based on inadequate therapeutic response (MMT-8 <10% improvement) or persistent inflammation, contingent upon favorable tolerability (absence of Common Terminology Criteria for Adverse Events Grade ≥2 adverse events, leukopenia, or hepatotoxicity).

Medication Parameter	Group 1 (n=17)	Group 2 (n=23)	p-value
Prednisolone Dosing (mg/day)			
Initial dose	17.9 ± 6.71	Not administered	—
Follow-up dose (24 weeks)	11.8 ± 5.04	Not administered	—
Absolute reduction	6.1 ± 3.42	—	—
Percentage reduction	34.08 ± 19.10%	—	—
Within-group comparison	t = 7.345^‡^	—	<0.0001
Mycophenolate Mofetil Dosing (g/day)			
Initial dose	Not administered	1.19 ± 0.259	—
Follow-up dose (24 weeks)	Not administered	1.69 ± 0.372	—
Absolute escalation	—	0.50 ± 0.228	—
Percentage escalation	—	42.02 ± 19.14%	—
Within-group comparison	—	t = 10.528^‡^	<0.0001

Safety profile and adverse event assessment

Systematic adverse event monitoring throughout the 24-week observation period documented favorable safety and tolerability profiles for both therapeutic regimens. Treatment-emergent adverse events occurred in 14 participants (35.0%) overall, with comparable incidence between Group 1 (n=6, 35.29%) and Group 2 (n=8, 34.78%), p=0.9732. Infectious complications included upper respiratory tract infections (n=5, 12.5%), one herpes zoster reactivation in Group 2 (4.35%), and one urinary tract infection in Group 2 (4.35%). All infectious episodes manifested as mild-to-moderate severity, resolved with standard antimicrobial therapy, and required no immunosuppressive medication discontinuation. Gastrointestinal disturbances (transient nausea, dyspepsia, or mild diarrhea) occurred in five participants (12.5%), predominantly in the mycophenolate cohort (n=4, 17.39% versus n=1, 5.88%, p=0.3668), representing Grade 1 severity with spontaneous resolution. Mild leukopenia (WBC 3000-3500/μL) developed in two mycophenolate recipients (8.70%), both asymptomatic and self-limiting. Corticosteroid-associated metabolic complications demonstrated higher incidence in Group 1, including hyperglycemia (23.53% versus 4.35%), cushingoid features (17.65% versus 0%), and pathological weight gain (17.65% versus 4.35%), with the composite metabolic adverse event rate significantly elevated in corticosteroid monotherapy recipients (41.18% versus 8.70%, p=0.0166). Transient asymptomatic transaminase elevation occurred in one participant per group (5.88% and 4.35%), both normalizing spontaneously. No serious adverse events, treatment discontinuations due to adverse events, or deaths occurred during the study period. Complete blood count and hepatic transaminases were assessed at baseline and each six-week follow-up interval throughout the 24-week observation period.

## Discussion

The present prospective, open-label, comparative observational study evaluated clinical outcomes between MMF combination therapy and corticosteroid monotherapy in IIM, documenting clinically meaningful muscle strength improvements, inflammatory biomarker reductions, and pulmonary function improvements in participants receiving MMF with lower cumulative corticosteroid exposure. However, critical methodological considerations require prominent acknowledgment: the statistically significant baseline MMT-8 score imbalance between treatment cohorts (79.7 versus 71.3, p=0.0017) reflects non-randomized physician-directed treatment allocation wherein patients with more severe disease presentation received MMF combination therapy. This baseline heterogeneity introduces confounding by indication that, despite analysis of covariance adjustment, limits definitive causal inference. These findings substantially corroborate observations documented by Nair et al. (2021), whose large South Asian cohort study (n=212) documented clinical improvements in muscle power and inflammatory marker reduction among IIM patients receiving MMF [12]. Our investigation demonstrated a mean MMT-8 score improvement of 12.5 points (17.53% increase, p=0.0006) in the MMF-treated group over 24 weeks, compared to 3.0 points (3.77% increase) in the corticosteroid monotherapy group. To address baseline severity differences, our primary inference utilized analysis of covariance adjusting for baseline disease severity, which demonstrated persistent between-group differences (adjusted mean difference: 4.23 points, 95% CI: 0.97-7.49, p=0.0127). However, participants with more severe baseline disease inherently possess greater absolute improvement potential, and unmeasured confounding factors associated with disease severity may influence treatment outcomes. These quantitative improvements align with the retrospective case series by Olivo Pallo et al. (2018), who documented clinically significant muscle strength restoration in seven patients with refractory systemic autoimmune myopathies receiving MMF [11]. Furthermore, Majithia and Harisdangkul (2005) initially described MMF as an effective alternative therapy for autoimmune inflammatory myopathy in their pioneering case series of eight patients, wherein six participants demonstrated clinical improvement with corticosteroid dose reduction ranging from 50% to 75% [10].

The inflammatory biomarker reduction patterns observed were comparable between MMF combination therapy and corticosteroid monotherapy, with both treatment approaches associated with substantial creatinine kinase reductions of approximately 32% from baseline concentrations. These findings parallel the comprehensive systematic review by Campanilho-Marques et al. (2025), which synthesized current treatment paradigms for idiopathic inflammatory myopathies and emphasized individualized therapeutic approaches combining corticosteroids with alternative immunosuppressive agents [5]. Notably, our investigation's inflammatory marker reduction trajectories closely mirror those documented by Hanaoka et al. (2019), who evaluated MMF treatment with or without calcineurin inhibitors in 23 patients with resistant inflammatory myopathy, demonstrating significant improvements with 74% achieving clinical improvement [17]. The comparable inflammatory biomarker reductions observed across both treatment modalities, with substantially lower corticosteroid doses in the MMF cohort, suggest that combination immunosuppressive approaches may achieve comparable biochemical disease control while requiring lower cumulative glucocorticoid exposure. This therapeutic principle receives additional support from Campochiaro et al. (2024), who evaluated the effectiveness and safety of MMF and rituximab combination therapy in 44 patients with idiopathic immune myopathies, documenting significant improvements, with 68% achieving a clinical response defined as ≥20% improvement in functional scales [18].

The pulmonary function improvements documented in participants with ILD (n=11) represent particularly clinically meaningful findings, given that ILD constitutes a major determinant of morbidity and mortality in IIM populations. Forced vital capacity improvements of approximately 12% observed align substantively with the comprehensive meta-analysis by Brown et al. (2021), which synthesized evidence regarding MMF's emerging role in ILDs across multiple systemic autoimmune conditions [19]. Furthermore, our findings corroborate the systematic review by Cassone et al. (2021) evaluating MMF efficacy and safety in rheumatic disease-related ILD, which established MMF as a therapeutically promising option with acceptable safety profiles [20]. The universal response rates documented in our radiographic assessment (100% in both cohorts experiencing either complete or partial resolution) provide supportive evidence for MMF's potential therapeutic benefit in pulmonary manifestations, though the modest diffusing capacity for carbon monoxide decline observed (approximately 7% reduction) warrants vigilant longitudinal surveillance for potential subclinical pulmonary parenchymal alterations [4].

The percentage improvement analysis (17.53% versus 3.77%, p=0.0004) and therapeutic convergence achieved at terminal assessment (83.8 versus 82.7, p=0.7893) suggest that MMF combination therapy may be associated with favorable therapeutic trajectories despite initial disease severity disparities. However, participants in Group 2 demonstrated substantially more severe muscle weakness at treatment initiation (baseline MMT-8 scores 71.3 versus 79.7, p=0.0017), reflecting systematic differences in treatment allocation. These findings contrast somewhat with the investigation by Edge et al. (2006), who documented MMF's clinical benefit in five patients with recalcitrant dermatomyositis, wherein all participants demonstrated clinical improvement but required substantially longer treatment durations (mean 14 months) to achieve optimal therapeutic response [21]. The accelerated therapeutic response trajectory observed in our 24-week investigation may reflect dose optimization protocols, with MMF escalation from 1.19 g to 1.69 g daily based on clinical response and tolerability profiles. The cutaneous manifestation response rates documented in our investigation (100% overall response with 40% complete resolution in Group 1, 36% in Group 2) align substantively with contemporary treatment paradigms synthesized by Barsotti and Lundberg (2018), who comprehensively reviewed current treatment options for myositis and emphasized the importance of individualized therapeutic strategies incorporating disease severity, subtype classification, and organ involvement patterns to optimize clinical outcomes [6].

The most substantial limitation of this investigation is the non-randomized treatment allocation design, which precludes definitive causal inference. The statistically significant baseline MMT-8 score imbalance (79.7 versus 71.3, p=0.0017) unequivocally demonstrates that treatment assignment was systematically influenced by initial disease severity, clinical phenotype, and prognostic factors - confounding by indication in observational comparative effectiveness research. Patients receiving MMF combination therapy represented a clinically distinct subpopulation with more severe baseline muscle weakness, potentially reflecting physician judgment that these patients required more aggressive immunosuppressive management. Consequently, the observed superior percentage improvement in the MMF group (17.53% versus 3.77%) may partially reflect greater potential improvement from lower baseline values (regression to the mean) rather than exclusively representing true therapeutic superiority. Although analysis of covariance adjustment for baseline disease severity partially mitigates observed confounding, this statistical approach cannot eliminate bias from unmeasured prognostic variables (disease duration, myositis-specific autoantibody profiles, genetic factors, concurrent medications) systematically associated with treatment allocation decisions. Therefore, these findings should be interpreted as hypothesis-generating observations establishing preliminary evidence that MMF combination therapy was associated with favorable clinical outcomes alongside lower corticosteroid dose requirements. Definitive conclusions regarding therapeutic superiority warrant confirmation through adequately powered randomized controlled trials with balanced baseline characteristics before incorporation into evidence-based clinical practice guidelines.

Clinical significance

The clinical implications of these findings provide hypothesis-generating observations that may inform future therapeutic investigation in contemporary IIM management, though definitive practice recommendations require validation through randomized controlled trials. The observed 34.08% corticosteroid dose reduction in monotherapy cohorts, combined with favorable clinical outcomes in MMF combination therapy participants receiving lower cumulative glucocorticoid doses, addresses the critical clinical challenge articulated by Curtis et al. (2006), whose population-based assessment (n=50,884) documented substantial adverse event burden associated with long-term glucocorticoid use [8]. Furthermore, Weinstein (2011) comprehensively detailed glucocorticoid-induced bone disease mechanisms, establishing that even modest corticosteroid doses substantially elevate fracture risk, thereby underscoring the clinical imperative for effective alternative immunosuppressive strategies [9]. These findings possess particular relevance for patient populations requiring prolonged immunosuppression or demonstrating corticosteroid intolerance, osteoporosis, glucose dysregulation, or other glucocorticoid-associated comorbidities, wherein MMF combination therapy was associated with lower corticosteroid dose requirements while maintaining comparable disease control measures. However, these observations require confirmation through adequately powered randomized trials before incorporation into evidence-based clinical practice guidelines.

Strengths of the study

This investigation's methodological strengths include a prospective design with systematic longitudinal assessment intervals facilitating comprehensive therapeutic trajectory evaluation. The utilization of validated outcome measures, including MMT-8 scoring with established inter-rater reliability (ICC 0.95), enhances measurement precision and reproducibility. Comprehensive multi-organ assessment encompassing muscular, pulmonary, dermatologic, and biochemical parameters provides holistic disease activity evaluation. The inclusion of participants with diverse IIM subtypes enhances external validity and clinical applicability across heterogeneous patient populations. Rigorous statistical methodology incorporating ANCOVA to adjust for baseline disease severity differences strengthens analytical rigor.

Limitations of the study

The non-randomized observational design with statistically significant baseline MMT-8 score imbalance (79.7 versus 71.3, p=0.0017) constitutes the most substantial methodological limitation. Group 2 participants demonstrated 8.4 points lower baseline muscle strength scores, reflecting confounding by indication whereby physician-directed treatment selection was systematically influenced by initial disease severity, clinical phenotype, and prognostic characteristics. Although ANCOVA adjustment for baseline disease severity partially mitigates observed confounding, this statistical approach cannot eliminate bias from unmeasured prognostic variables systematically associated with treatment allocation decisions (disease duration, prior treatment exposures, myositis-specific autoantibody profiles, genetic factors, and comorbid conditions). Therefore, observed treatment effects may be confounded by systematic differences between groups beyond initial disease severity alone. Consequently, these findings should be interpreted as hypothesis-generating observations rather than definitive evidence of treatment superiority. The modest sample size (n=40) constrains statistical power for detecting subtle between-group differences and precludes comprehensive subgroup analyses stratified by disease subtype, organ involvement, or autoantibody profiles. The 24-week observation period provides adequate temporal resolution for short-term therapeutic response assessment but insufficient duration for evaluating long-term treatment efficacy, adverse event profiles, disease relapse rates, or cumulative corticosteroid exposure effects. The single-center design potentially limits generalizability to broader geographic populations. Convenience sampling methodology may introduce recruitment bias. The absence of standardized quality-of-life assessments limits comprehensive evaluation of patient-reported outcomes beyond objective clinical parameters.

Recommendations

Future investigations should consider multicenter collaborative frameworks to enhance sample size adequacy and generalizability. Extended observation periods (≥12 months) would facilitate long-term outcome assessment, including disease relapse rates and sustained therapeutic response maintenance. Incorporation of standardized quality-of-life instruments would capture treatment impact on functional status and disability indices. Most critically, adequately powered randomized controlled trials with balanced baseline characteristics are essential to establish definitive causal inference regarding MMF's therapeutic efficacy and steroid-sparing capacity before incorporation into evidence-based clinical practice guidelines.

## Conclusions

This prospective observational study provides preliminary evidence that MMF combination therapy demonstrates potential therapeutic benefit in IIM management. The MMF regimen achieved statistically significant muscle strength improvements, inflammatory biomarker reductions comparable to corticosteroid monotherapy, and clinically meaningful pulmonary function enhancement in participants with ILD. Critically, MMF combination therapy enabled substantially reduced cumulative corticosteroid exposure compared to prednisolone monotherapy, potentially mitigating glucocorticoid-associated metabolic complications including osteoporosis, glucose dysregulation, and immunosuppression-related infectious risks. However, fundamental methodological limitations temper definitive conclusions. The non-randomized treatment allocation with significant baseline disease severity differences introduces confounding by indication, precluding definitive causal inference regarding treatment superiority. These hypothesis-generating observations warrant confirmation through adequately powered, multicenter randomized controlled trials with balanced baseline characteristics. Pending confirmatory investigation, MMF represents a therapeutically promising immunosuppressive strategy meriting consideration for patients requiring prolonged immunosuppression or demonstrating corticosteroid intolerance, with potential for reduced glucocorticoid-associated morbidity in routine clinical practice.
